# Focal therapeutic conditions matter: How energy affects antigen presentation and T cell activation

**DOI:** 10.1002/btm2.70152

**Published:** 2026-05-27

**Authors:** Minhan Jiang, Qi Shao, Joseph Vallin, Yoji Shimizu, Brandon Burbach, Samira Azarin, John Bischof

**Affiliations:** ^1^ Department of Mechanical Engineering and Department of Biomedical Engineering University of Minnesota Minneapolis Minnesota USA; ^2^ Department of Neurosurgery University of Minnesota Minneapolis Minnesota USA; ^3^ Department of Chemical Engineering and Material Science University of Minnesota Minneapolis Minnesota USA; ^4^ Department of Laboratory Medicine and Pathology University of Minnesota Minneapolis Minnesota USA; ^5^ Center for Immunology, University of Minnesota Minneapolis Minnesota USA; ^6^ Present address: Department of Mechanical Engineering University of Minnesota Minneapolis Minnesota USA

**Keywords:** antigen presentation, cryoablation, immunomodulation, irreversible electroporation, T cell activation, thermal therapy, tumor ablation

## Abstract

Energy‐based minimally invasive cancer focal therapies (FTs), including thermal ablation (Heat), cryoablation (Cryo), and irreversible electroporation (IRE), can induce immunomodulatory effects and can synergize with immunotherapy to engage the immune system for systemic and long‐term antitumor response. However, the immunogenicity of each FT can vary based on treatment specifics, and the mechanisms underlying these differences are not fully understood. We employed a model antigen (ovalbumin, OVA) and an in vitro platform to investigate the upstream processing steps that initiate immune priming. This platform enabled quantitative assessment of antigen release, antigen presentation, dendritic cell (DC) activation, antigen‐specific T cell proliferation and the generation of damage‐associated molecular patterns (DAMPs), cytokines and chemokines. Both antigen‐specific signaling and T cell response are highly dependent on the focal therapeutic conditions applied to the cancer cells. Cryo resulted in the highest release of protein, OVA antigen, ATP, and HMGB1, followed by IRE and Heat. Despite not yielding the most antigen, IRE‐treated B16‐OVA elicited the most DC activation and OVA‐specific T cell proliferation. Dose–response analysis using OVA protein and SIINFEKL peptide confirmed a positive correlation between antigen availability and T cell response. We also observed substantial adjuvant effects that enhance the immune processing and T cell response by stimulus in cell lysates independently of antigen quantity. Specifically, IRE‐treated B16 cell lysates induced the most significant improvement in dendritic cell (DC) activation and T cell proliferation compared to Heat and Cryo when paired with equal amounts of antigen. Our findings position IRE as the most immunogenic FT condition in our system. This study highlights the critical interplay between antigen release, adjuvant signals, and immune processing in shaping FT‐driven antitumor immunity.

## Graphical abstract

Irreversible Electroporation (IRE) physically destroys tumor cells, triggering a powerful “danger signal” in the body. This study demonstrates that IRE not only kills the tumor but efficiently releases tumor‐specific antigens, which prime dendritic cells to activate a robust, targeted T‐cell immune response against the cancer.

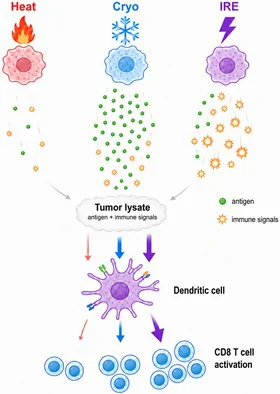

**Translational Impact Statement**

Energy‐based tumor ablative techniques can induce immunomodulatory effects to engage the immune system for systemic and long‐term disease control. However, the immunogenicity of each technique can vary based on treatment specifics, and the underlying mechanisms are not fully understood. Using an in vitro B16 model and a model antigen, our findings revealed that irreversible electroporation (IRE) emerged as the most immunogenic treatment, characterized by inducing the most significant improvement in dendritic cell activation and T cell proliferation compared to cryoablation and thermal ablation.

## 1 INTRODUCTION

Focal therapies (FTs) are minimally invasive cancer treatments that employ energy‐based techniques to selectively target solid tumors while preserving healthy tissue with fewer side effects.^1^ Techniques used for tumor destruction include thermal ablation (Heat), cryoablation (Cryo), and irreversible electroporation (IRE). Besides tumor ablation, the immunogenic effects are gaining more attention for their importance in combatting local recurrence and systemic metastasis.^2^, ^3^, ^4^, ^5^ To this end, combinatorial approaches incorporating FT with various forms of immunomodulators and immunotherapies (especially immune checkpoint inhibitors, ICIs) are being investigated and are under clinical trial.^1^, ^6^, ^7^, ^8^, ^9^, ^10^ This highlights the need for better comprehension of the interaction between physical destruction of cancer cells and the immune response, particularly the immunomodulatory effect by FT and the following evoked anti‐cancer immunity.

While combinatorial approaches are more commonly used to achieve more systemic and durable cancer control, several important questions remained unanswered, such as how to determine the best choice of FT in a combination regimen to optimize treatment efficacy is usually not straightforward. This is partly due to the fact that clinical decisions are typically based on tumor location, size, and disease stage.^1^ The optimal dosing and timing for combination therapy remain largely unknown. To shed light on these questions, direct comparative analysis of the immunogenicity by various FT modalities is needed. Following exposure of tumor to lethal energy fields, immunogenicity pertains to the capacity of tumor cells and associated molecules to induce an immune reaction, leading to antigen recognition, immune cell activation, and memory formation. This multifactorial process highlights the importance of thoroughly understanding how immunogenicity correlates with treatment conditions and their underlying mechanisms.

To further understand how different FT modalities modulate the anti‐cancer immunity, the OVA model antigen was used to study potential factors that mediate the immunological effects of FTs, including antigen release, generation of damage‐associated molecular patterns (DAMPs), cytokines and chemokines, antigen presentation and activation of antigen‐specific T cells. We found that T cell proliferation and DC activation following IRE treatment of B16‐OVA cells were dramatically more robust compared to Cryo or Heat, while Cryo released the most protein, antigen, adenosine triphosphate (ATP) and high mobility group box 1 (HMGB1). The profiles of cytokine and chemokine release from tumor cells following different treatments exhibit variation. Importantly, further analysis revealed that immune processing and T cell responses are shaped not only by antigen, but also by other stimuli present in cell lysates.

Our study demonstrates that the immunogenic response and immune priming can be engineered by cancer cell treatment, which involves many factors including antigen release (amount and accessibility) and the generation of DAMPs, cytokines, and chemokines. Additionally, this in vitro platform could serve as a useful tool for optimizing focal therapy parameters to achieve optimal T cell activation, as studies have shown that energy‐cell interaction can dramatically influence immunogenic cell death.^11^ These mechanistic insights will help us engineer focal therapies to be better in situ tumor vaccines and serve as the foundation for optimizing combinatory strategies with immunotherapy (including ICIs) to address local recurrence and metastasis.

## 2 MATERIALS AND METHODS

### 2.1 Antigen model

Ovalbumin (OVA) is a T cell‐dependent antigen commonly used as a model protein for studying antigen‐specific immune responses in mice. OVA contains two major immunodominant epitopes for eliciting T cell responses: the class I‐restricted OVA 257‐264 (SIINFEKL) and class II‐restricted OVA 323–339. The SIINFEKL peptide is presented by the (H‐2K^b^) MHC‐1 molecule^12^ and is widely used to monitor CD8+ cytotoxic T cell response.^13^, ^14^ In contrast, OVA 323–339 encompasses an allergenic and antigenic epitope^15^ and is used extensively to study the nature of class II MHC–peptide binding and CD4+ T cell activation.^16^, ^17^ By utilizing the OVA model, we can quantitatively monitor the immune processing of a specific antigen.^18^ Additionally, ovalbumin is widely used as a model antigen, given that cancer cells can be engineered to express it consistently.^19^, ^20^ The commercially available purified OVA, along with assays and kits for quantifying immune responses, makes OVA a highly convenient and cost‐effective model antigen for cancer immunology research.

### 2.2 In vitro platform

In this study, we focus on the CD8 T cell pathway considering cytotoxic T cells are the most powerful effectors in the anti‐cancer immune response and form the backbone of current successful cancer immunotherapies.^21^ In tumors, numerous antigens, complex physiochemical conditions of antigens, cell‐to‐cell interactions, and intricate cell migration patterns are present. However, by utilizing the in vitro assessment platform with the OVA model, we can track the immune processing of a specific antigen and quantitatively evaluate immune response, as shown in Figure 1.

**FIGURE 1:**
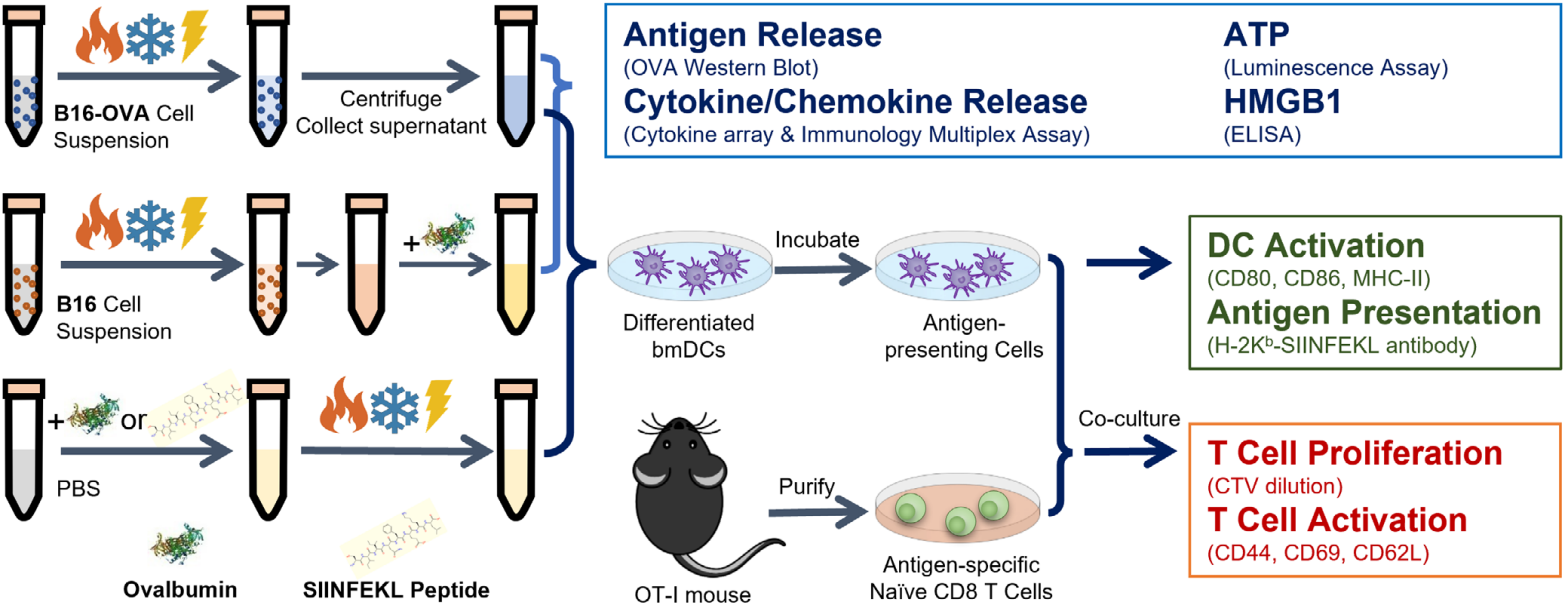
Schematic of in vitro treatments and assessments. B16 and B16‐OVA cell lines were exposed to various focal treatment conditions, including thermal ablation (Heat), cryoablation (Cryo), and irreversible electroporation (IRE). The model antigen (OVA) was used to study key determinants of the immunological effects of focal therapies including antigen release, generation of DAMPs/cytokines/chemokines, antigen presentation, and activation of antigen‐specific T cells. Heat: 50°C and 30 min for cells, 90°C and 30 min for OVA protein and SIINFEKL peptide; Cryo, −80°C for 30 min; IRE, 99 50‐μs pulses at 1250 V/cm with 1 Hz intervals.

This study offers an in vitro platform to characterize the antigen‐specific immune response, quantitatively assess immunogenic response by cancer cell treatments, and analyze various immune factors in a relatively simple system. Additionally, this platform enables standardization across different studies, facilitating result comparisons and the derivation of meaningful conclusions.

### 2.3 Cell culture and treatments

The melanoma cell line B16‐F10 (B16) was obtained from the American Type Culture Collection (ATCC). For Authentication and quality control, ATCC has developed a database of Short Tandem Repeat (STR) profiles for mouse cell lines. They generate an STR profile for each new lot of B16‐F10 cells (RRID:CVCL_0159) and compare it against the baseline profile of the original stock to ensure its identity and that it is free from mycoplasma contamination. Cells were cultured in Dulbecco's Modified Eagle Medium (DMEM, Gibco) supplemented with 10% Fetal Bovine Serum (FBS, Gibco), L‐Glutamine (Gibco) and Penicillin–Streptomycin (Gibco), incubated in tissue‐culture treated plastic flasks at 37°C with 5% CO_2_ and split every 2–3 days using Trypsin–EDTA (0.05% and 0.53 mM, Gibco). B16‐OVA‐GFP cells (B16‐OVA) were obtained from Brian Fife (University of Minnesota). Briefly, B16 lines were transduced with retroviral constructs containing the membrane‐bound form of OVA and GFP, and single‐cell subclones of high GFP expression were selected. Transduction was confirmed via GFP expression by flow cytometry. B16‐OVA cells were cultured in RPMI‐1640 medium, supplemented with 10% FBS, 2 mM L‐glutamine, 10 mM HEPES, 1 mM sodium pyruvate (Gibco), 1× Non‐Essential Amino Acids (NEAA, Gibco), 28 μM 2‐Mercaptoethanol (Gibco), 100 U/mL penicillin, and 100 μg/mL streptomycin. Cells were used within four passages of receipt and were free from mycoplasma contamination. Cells were detached using 0.05% Trypsin–EDTA when >85% confluence.

For in vitro cell treatment and assessments, B16‐OVA or B16 cells were trypsinized, centrifuged, washed twice and resuspended in PBS. For Heat treatment, cells in suspension were heated in a water bath at 50°C for 30 min before passively cooling to room temperature. For Cryo treatment, the cell suspension was frozen to −80°C in a methanol bath (equilibrated in −80°C freezer) for 30 min then thawed at room temperature. For IRE treatment, the prepared cell suspension was pipetted into an electroporation cuvette between the two plate electrodes. The cuvette was connected to an electric pulse generator (ECM 830, BTX) when the cells were exposed to an electric field at 1250 V/cm for 99 pulses with a pulse duration of 50 μs on 1 Hz. The IRE parameters (99 pulses, 50 μs, 1250 V/cm) were chosen based on established protocols for in vitro B16‐F10 ablation to ensure complete cell death without inducing thermal damage.^22^, ^23^, ^24^, ^25^ The positive control condition for cell lysis (LN_2_ cycling) involved employing three freeze–thaw cycles in LN_2_ followed by passive thaw to room temperature. All treatment conditions were previously described and designed for complete cell death.^23^ Following all treatments, the cell suspension sample was immediately subjected to low‐speed centrifugation (200×g for 10 min) to collect supernatant, which was defined as cell lysate. The cell lysates were aliquoted and stored at −80°C for further analysis.^26^, ^27^ On the day of analysis, cryopreserved lysate supernatants were thawed and homogenized by passing through a 30G syringe 5 times.

Whole Ovalbumin protein (Sigma A2512, lyophilized powder, ≥98% [agarose gel electrophoresis]) and the associated OVA peptide SIINFEKL (Anaspec AS‐60193) were exposed to the same FT treatment conditions except Heat. For the purpose of complete denaturation, pure protein and peptide solutions in PBS were heated in a water bath at 90°C for 30 min before passively cooling to room temperature.

### 2.4 Experimental mice

C57BL/6J mice (RRID:IMSR_JAX:000664, 8–12 weeks) were obtained from Jackson Laboratory (Bar Harbor, ME) and were used for bone marrow extraction. Transgenic OT‐I mice (Strain C57BL/6‐Tg(TcraTcrb)1100Mjb/J from The Jackson Laboratory, RRID:IMSR_JAX:003831), where the CD8+ T cells bear a genetically encoded receptor against ovalbumin residues 257–264 (SIINFEKL),^13^ were bred and maintained at the Yoji Shimizu lab (University of Minnesota) as previously described^28^ and were used as a source of antigen‐specific T cells for in vitro T cell activation analysis. Each biological replicate used cells from a separate mouse. Mice from both sexes were used, and no difference in biological response was observed.

### 2.5 Isolation of antigen‐presenting cells (APCs) and antigen‐specific T cells

To generate bone‐marrow derived DCs, C57BL/6J mice were euthanized by cervical dislocation under isoflurane anesthesia. Tibia and femurs of mice were isolated and rinsed with T cell proliferation media (TCPM, which consists of RPMI 1640, 10% FBS, 1% Penicillin/Streptomycin, 1 mM L‐Glutamine, 10 mM HEPES, 1 mM sodium pyruvate, 1× NEAA, and 25 μM 2‐Mercaptoethanol).^23^ Bone marrow was flushed from the tibia and femurs with TCPM, followed by drawing through the syringe twice to make single cell suspensions. Then the suspension was filtered through a 70 μm filter, rinsed, and subjected to blood cell lysis by ACK lysis buffer. Bone marrow‐derived cells were then incubated on non‐tissue culture treated plastic plates at 2.0 × 10^6^ cells/ml in TCPM in the presence of 100 ng/mL FMS‐like tyrosine kinase 3 ligand (Flt3L) for 9 days to promote differentiation of dendritic cells (DCs) into antigen presenting cells (APCs).^29^, ^30^ The purity and phenotype of the DCs were determined by flow cytometry for a range of surface lineage and phenotypic markers including CD8a, CD11c, CD11b, CD103, XCR1 and CD172a (SIRPα) (Figure S1).

To isolate antigen‐specific T cells, lymph nodes (LNs) and spleens were harvested from OT‐I transgenic mice and washed with TCPM. Subsequently, the tissues were dissociated and passed through a 70 μm cell strainer to obtain single‐cell suspensions. Naïve OT‐I T cells were purified by negative selection using a CD8 T cell isolation kit (EasySep, STEMCELL Technologies, Canada) according to the manufacturer's instructions, and the purity was confirmed by flow cytometry analysis (>95%). Purified OT‐I T cells were suspended in PBS with 10% FBS and then labeled with 5 μM Cell‐Trace Violet (CTV) dye for 15 min at 37°C as previously described^23^ prior to incubation with DCs.

### 2.6 DC and T cell activation assay

The workflow for assessing DC and T cell activation following incubation with tumor cell lysates or antigen solution is illustrated in Figure 2. Flt3L differentiated bone marrow‐derived DCs (bmDCs) were released using cold 3 mM EDTA, washed, and incubated overnight at 37°C in TCPM with B16 or B16‐OVA derived lysates generated as described above, or with OVA or peptide (SIINFEKL) solution, to allow antigen processing and presentation. DCs were collected and stained for flow cytometry in FACS buffer (HBSS containing 0.1% sodium azide and 2% bovine serum) containing a cocktail of fluorescently conjugated antibodies. The activation of DCs was assessed by the expression of activation markers (CD80, CD86, and MHC‐II), and an anti‐mouse H‐2K^b^ bound to SIINFEKL antibody (clone 25‐D1.16, Biolegend)^31^ was used to track the antigen presentation by quantifying the percentage of MHC I‐SIINFEKL complexes and/or the fluorescence intensity of the bound antibody. The flow cytometry gating strategy for DC activation is shown in Figure S2.

**FIGURE 2:**
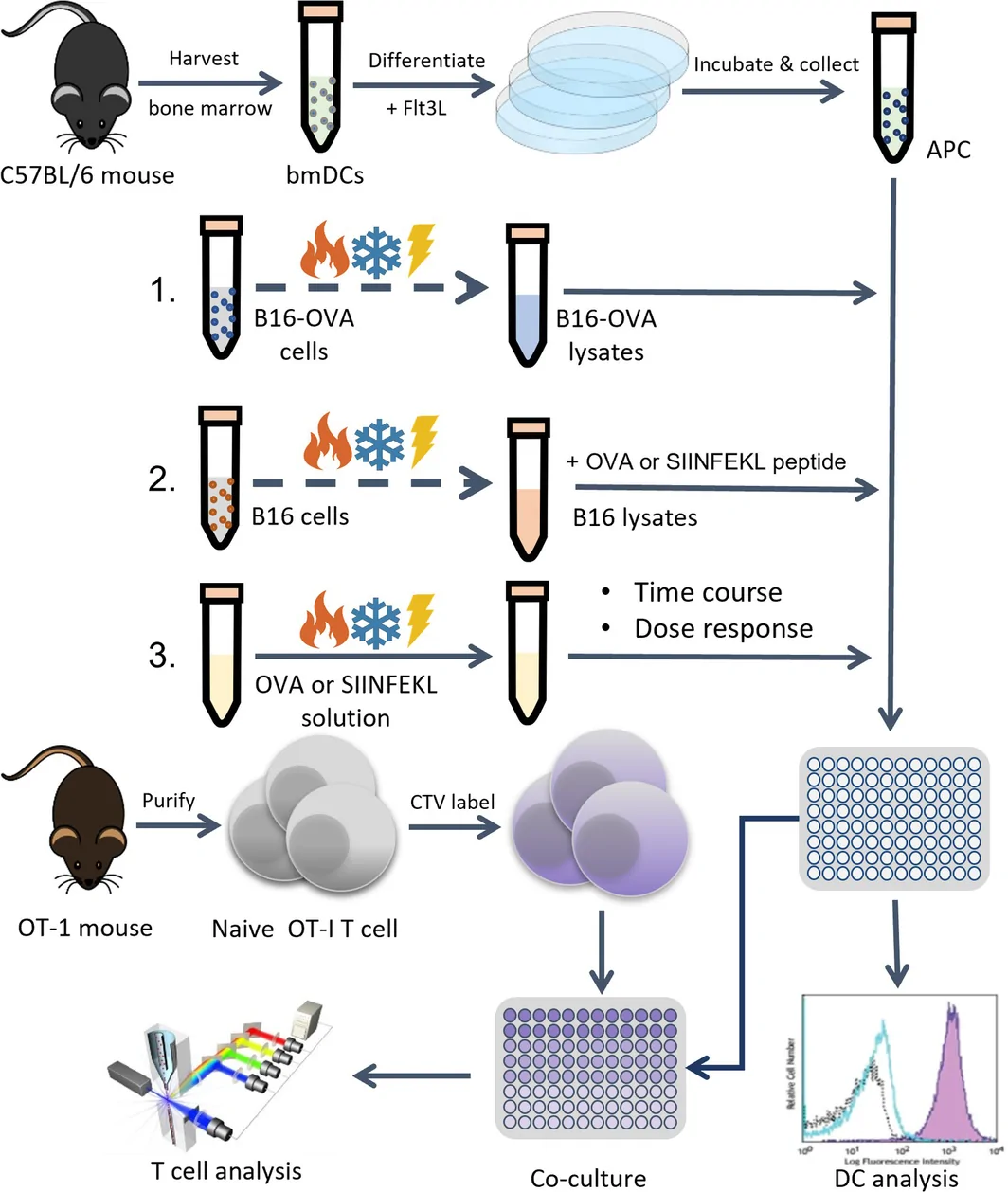
In vitro antigen presentation assay and antigen‐specific T cell response assay. Bone marrow‐derived DCs (bmDCs) are differentiated into antigen presenting cells (APCs) and incubated with B16/B16‐OVA lysates, OVA or peptide (SIINFEKL) solution to allow antigen processing and presentation. DC analysis includes activation (by surface markers CD80, CD86, and MHC‐II) and antigen presentation (MHC I‐SIINFEKL complexes). T cell analysis includes proliferation (by CTV dilution) and T cell activation (by surface markers CD69, CD44, and CD62L).

For T cell response assay, antigen‐pulsed APCs and CTV‐labeled naïve OT‐I T cells were cocultured in 96‐well TC‐treated flat bottom plates in the presence of TCPM. At various time points (i.e., 6 h for early priming/activation and 72 h for proliferation), T cells were collected from the plates and stained with fluorescently conjugated antibodies for 30 min on ice for flow cytometry analysis. T cell activation was assessed by upregulation of CD69 and CD44, and downregulation of CD62L on cell surface.^32^ The flow cytometry gating strategy for T cell activation is shown in Figure S3. T cell proliferation was assessed by defining the fraction of OT‐I T cells (CD8^+^Thy1.1^+^MHCII^−^) with diluted CTV. Non‐responding or naïve T cells retained high CTV intensity while actively proliferating T cells exhibit diluted CTV stain.

### 2.7 Flow cytometry

Aliquots of single‐cell suspensions were stained for flow cytometry in a 50 μL FACS buffer containing a cocktail of fluorescently conjugated antibodies at 1:200 dilutions or following manufacturer's instructions. CD69‐PE (clone H1.2F3), CD11c‐PE (clone N418), CD90.1 (Thy‐1.1)‐Pacific Blue (clone OX‐7), CD172a (SIRPα)‐PerCP/Cyanine5.5 (clone P84), MHC Class II (I‐A/I‐E)‐FITC (clone M5/114.15.2), CD172a (SIRPα)‐Brilliant Violet 510 (clone P84), XCR1‐Brilliant Violet 421 (clone ZET), XCR1‐Brilliant Violet 785 (clone ZET), MHC Class II (I‐A/I‐E)‐Brilliant Violet 510 (clone M5/114.15.2), CD8a‐APC (clone 53‐6.7), MHC Class II (I‐A/I‐E)‐PE/Cyanine7 (clone M5/114.15.2), CD86‐PE/Cyanine7 (clone GL‐1), CD45‐Alexa Fluor 700 (clone 30‐F11), CD11c‐Brilliant Violet 650 (clone N418) were obtained from BioLegend (San Diego, CA). Ghost Dye Violet 510 viability stain (1:400), CD8a‐PE (clone 53‐6.7), CD8a‐PerCP‐Cyanine5.5 (clone 53‐6.7), CD44‐FITC (clone IM7), CD86 (B7‐2)‐FITC (clone GL‐1), CD62L (L‐Selectin)‐APC (clone MEL‐14), CD45.1‐APC (clone A20), CD45.2‐APC (clone 104), and CD11c‐PE‐Cyanine7 (clone N418) were obtained from Tonbo Biosciences (San Diego, CA). Samples were analyzed on an LSRII or Fortessa digital flow cytometer (BD Biosciences), and the data processed with FlowJo (v10) software.

### 2.8 Protein and antigen release assessment

Total protein release was measured with Pierce bicinchoninic acid (BCA) Protein Assay Kit (Thermo Scientific) according to the manufacturer's instructions. In this study, we define protein release as the content of protein within the lysate.

Ovalbumin in the lysate supernatants was measured via western blot. Briefly, for each focal treatment method, 15 μL supernatant was mixed with 5 μL of 4× Laemmli protein sample buffer (Bio‐Rad) containing 10% β‐mercaptoethanol (MilliporeSigma) and then boiled at 97°C for 5 min using a C1000 Touch™ thermal cycler (Bio‐Rad). The mixture was loaded into a 4–15% Mini‐PROTEAN TGX Precast 12‐well Protein Gel (Bio‐Rad). The gel was run at 200 V for 31 min using a 2‐Gel Tetra and Blotting Module (Bio‐Rad) in 1× Tris/Glycine/SDS buffer (Bio‐Rad). Proteins were transferred to an Immun‐Blot PVDF membrane (Bio‐Rad) at 100 V for 1 h in ice cold 1× Tris/Glycine buffer (Bio‐Rad) containing 20% methanol (MilliporeSigma). The membrane was then blocked in 5% milk in 1× Tris buffered saline Tween‐20 (TBST) buffer (Thermo Scientific). It was then incubated with a primary ovalbumin polyclonal antibody (Invitrogen, PA1‐196) diluted 1:1000 in 5% bovine serum albumin (MilliporeSigma) in TBST buffer at 4°C overnight. After washing three times with TBST, the membrane was incubated with the secondary HRP‐linked goat anti‐rabbit IgG antibody (Cell Signaling, 7074S) in 5% milk in TBST for 1 h at room temperature. After washing three times with TBST, the membrane was incubated with 1× Super Signal West Pico Plus Chemiluminescent substrate for 30 s and the chemiluminescence was measured with a ChemiDoc Touch Imaging System (Bio‐Rad). After imaging, the membranes were washed twice in TBST and then placed in a staining solution of 0.1% Coomassie R‐350 in a 1:1 methanol and water mixture for 1 min. The membranes were lightly drained and then placed in a destaining solution (a 1:5:4 mixture of glacial acetic acid, ethanol, and water, respectively) for 20 min. After allowing them to fully air dry, the membranes were imaged using the ChemiDoc Touch imaging system to measure the total protein in each lane.

### 2.9 DAMP, cytokine and chemokine measurement

For ATP release measurement with the ATPlite Luminescence ATP Detection Assay (PerkinElmer), fresh B16‐OVA cell lysates were used. Immediately after spinning down the treated cells, the Lysate Buffer from the ATPlite kit was added to the collected supernatant in a 1:2 ratio to prevent premature ATP degradation. The rest of the assay was performed according to the manufacturer's instructions. HMGB1 was measured with an HMGB1 ELISA kit (Novus Biologicals) according to the manufacturer's instructions. Cytokines and chemokines were measured with Mouse Cytokine Array Panel A (R&D Systems) and MILLIPLEX MAP Mouse Cytokine/Chemokine Magnetic Bead Panel (MilliporeSigma) according to the manufacturer's instructions.

### 2.10 Statistics

GraphPad Prism software version 10 was used to perform statistical analysis. Three batches (biological replicates) of B16 and B16‐OVA cells were independently used for assessments and assays to confirm the results. For each biological replicate, averages were taken from at least two technical replicates. All data are expressed as mean ± SD unless indicated otherwise. Statistical differences between groups were evaluated by using either the unpaired t test with Welch's correction, ordinary one‐way or two‐way ANOVA multiple comparisons depending on the data. In all cases, *p* values are: *p < 0.05; **p < 0.01; ***p < 0.001; ****p < 0.0001. A *p* value of <0.05 was considered statistically significant.

## 3 RESULTS

### 3.1 IRE‐treated B16‐OVA cells led to the highest proliferation of antigen‐specific T cells and strongest DC activation

The response of SIINFEKL‐specific CD8 T cells (OT‐I T cells) after co‐culture with DCs pre‐pulsed with treated B16‐OVA cell lysates was assessed by an activation and proliferation assay (Figure 3a). The extent of T cell proliferation was quantified by the percentage of cells with reduced CTV intensity (CTV low), defined as the T cell proliferation percentage. Figure 3b shows the percentage of proliferated OT‐I T cells (CTV low) in CD8^+^Thy1.1^+^MHCII^−^ gated T cells under different conditions. In the absence of antigen, the T cells do not proliferate (0%) (Figure S4). In contrast, a low concentration of SIINFEKL peptide (0.1 pg/mL) resulted in priming of all (>99.9%) antigen‐specific T cells, indicating the sensitivity of the SIINFEKL‐specific T cell activation assay (Figure S4). Subsequently, proliferation percentages were assessed across the titration of cell lysates from each focal therapeutic condition. The LN_2_ freeze–thaw cycling condition (positive control) yielded the maximum proliferation index from the B16‐OVA cell lysates (98 ± 0.56%) at 5 × 10^5^ cell equivalents, followed by IRE (91.5 ± 2.7%), Cryo (59.8 ± 19.1%), and Heat (51.3 ± 6.7%). At higher cell equivalent numbers, there was a consistent trend of increased T cell proliferation for all focal therapeutic conditions (Figure 3b). However, IRE promoted a substantially higher T cell proliferation index than Cryo or Heat over the same cell equivalent range (5 × 10^3^–5 × 10^5^ cells). Additionally, Cryo modestly outperformed Heat at higher cell equivalents, although without significant statistical differences (Figure 3b). The comparisons among Heat, Cryo, and IRE treatments are listed in Table S1.

**FIGURE 3:**
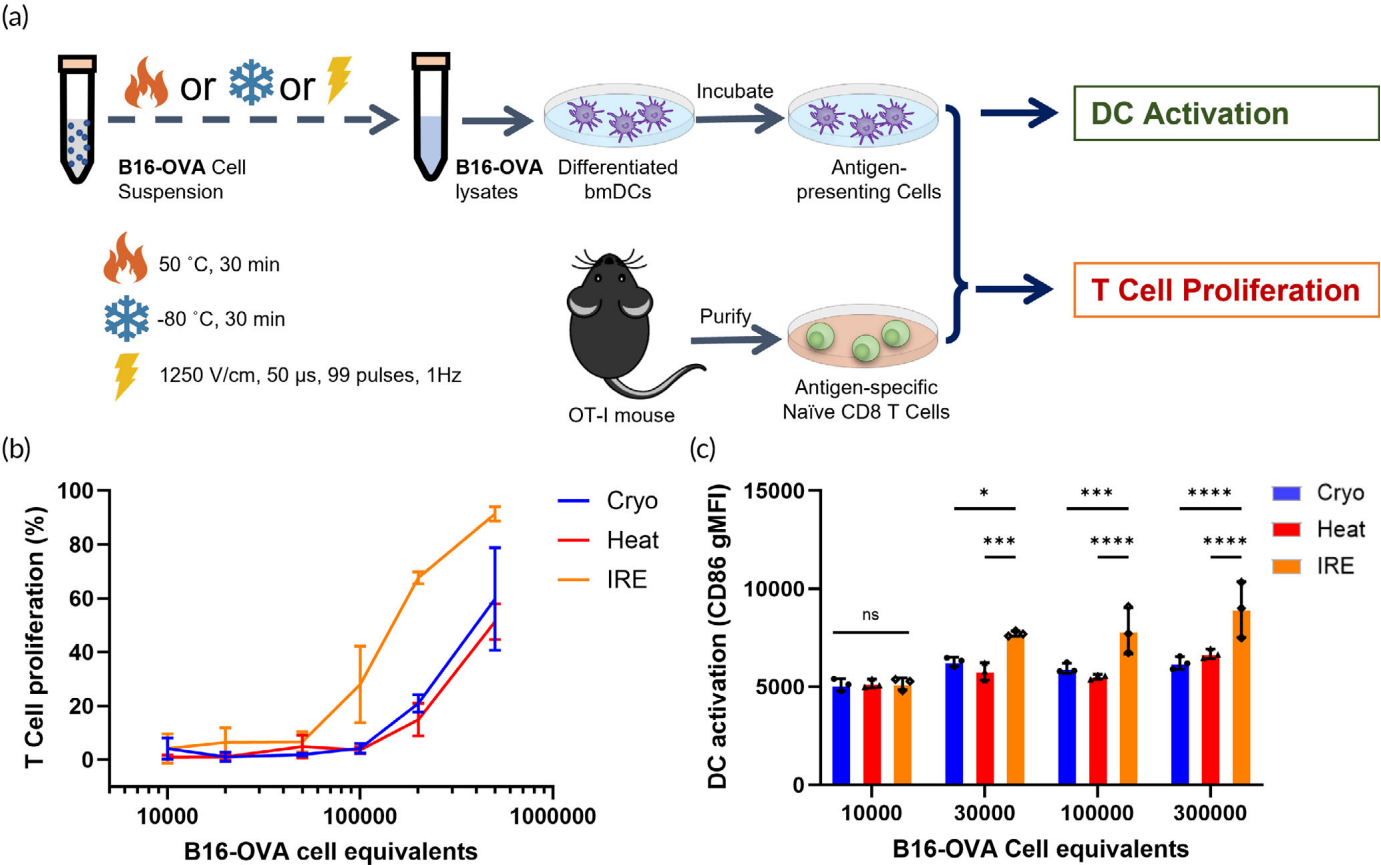
T cell and DC response to B16‐OVA cell lysates after focal therapy condition exposure. (a) Schematic of characterization assays. (b) The proliferation percentage of OT‐I T cells was quantified at various cell equivalents. The ratio of DCs to T cells was 3:1. (c) Expression of DC activation marker (CD86) on bone marrow‐derived DCs after incubation with treated B16‐OVA cell lysates for 24 h at various cell equivalents. Expression was quantified via geometric mean fluorescence intensity (gMFI). Data are presented as mean ± standard deviation (SD). Significance of differences was determined using 2‐way ANOVA multiple comparisons. *p < 0.05, **p < 0.01, ***p < 0.001, ****p < 0.0001. n.s. not significant.

The activation status of DCs was further characterized following stimulation with lysates from treated B16‐OVA cells. Flow cytometry analysis revealed that IRE‐treated cell lysates induced the highest expression of CD86 on DCs compared to other treatments at various cell equivalents, as indicated by the geometric mean fluorescence intensity (gMFI) (Figure 3c). The expression levels of other activation markers (CD80, MHCII) are depicted in Figure S5, revealing no significant differences but demonstrating a consistent trend at some cell equivalents. Taken together, these results suggest that the method of focal treatment applied to cells can influence the response of DCs and T cells, and IRE is shown to achieve superior immune priming effects compared to Cryo and Heat.

### 3.2 Cryo‐treated B16‐OVA cells had the highest antigen, ATP and HMGB1 release

Lysates collected from B16‐OVA cells after exposure to all treatment conditions were evaluated for total protein release using a BCA assay and for antigen release by western blot (Figure 4a). The highest protein release was observed after the LN_2_ freeze–thaw cycling condition (positive control) (2340 ± 582 μg/mL), followed by Cryo (1912 ± 373 μg/mL), IRE (1024 ± 173 μg/mL), and Heat (465 ± 175 μg/mL) treatments (Figure 4b). To assess relative antigen (OVA) release, the OVA expression normalized to the LN_2_ condition was quantified. Cryo resulted in the highest OVA release (126.4 ± 18.9%), followed by IRE (44.2 ± 10.1%) and Heat (5.4 ± 4.0%) compared to LN_2_ control (Figures 4c and S6). These results demonstrate a substantial difference in the total protein release across the evaluated focal therapy conditions. The release of OVA antigen follows a similar trend to that of total protein, with the highest levels observed after Cryo, followed by IRE, and the lowest by Heat.

**FIGURE 4:**
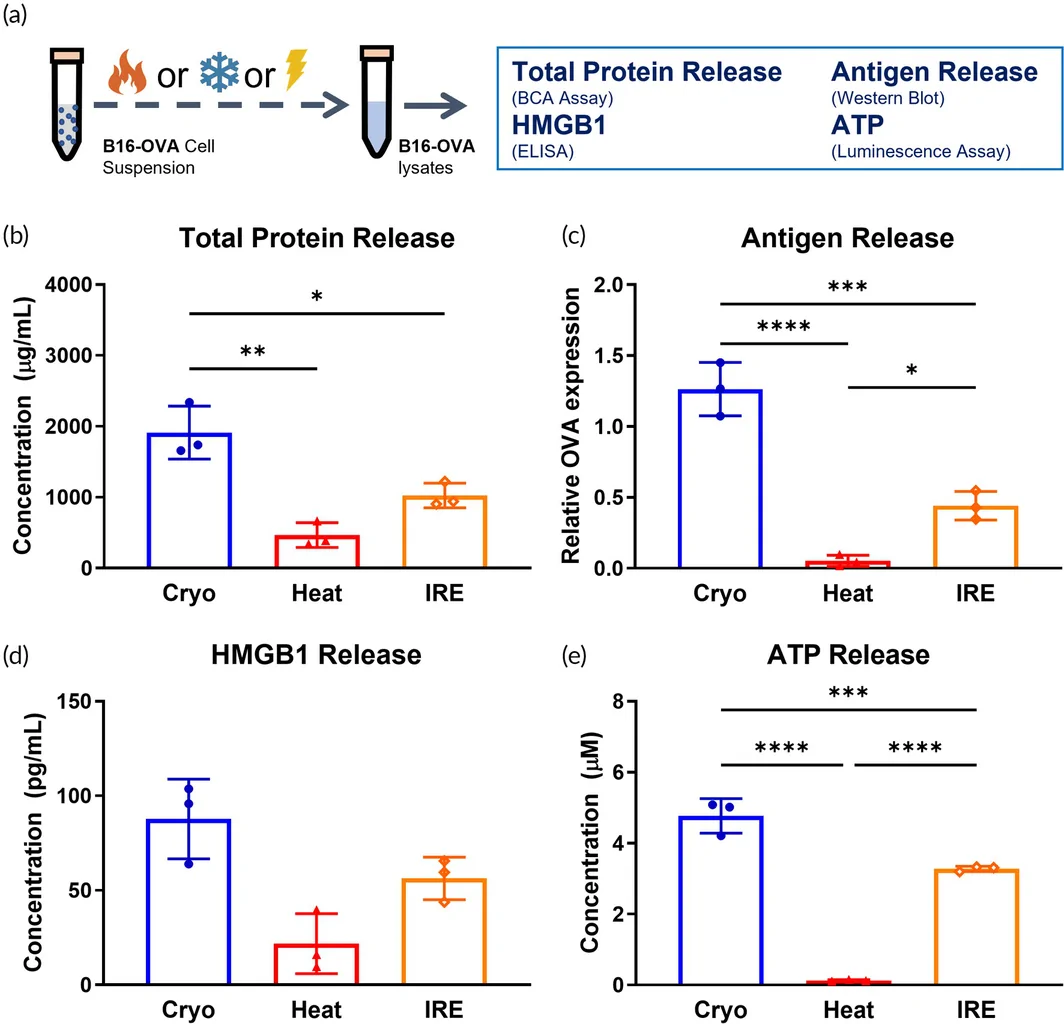
Quantification of total protein, antigen, HMGB1 and ATP release from B16‐OVA cells by different focal therapies. (a) Schematic of characterization assays. (b) Comparison of the total amount of protein released from the B16‐OVA cells into lysates after different focal treatments using the BCA assay. (c) Quantification of relative antigen release by different focal therapies compared to the LN_2_ control using western blot. (d) HMGB1 concentrations in cell supernatants following different focal therapies, analyzed using an enzyme‐linked immunosorbent assay (ELISA). (e) ATP concentrations in cell supernatants following different focal therapies, measured using a bioluminescence assay. Data are presented as mean ± standard deviation (SD). The significance of differences was determined using ordinary 1‐way ANOVA. *p < 0.05, **p < 0.01, ***p < 0.001, ****p < 0.0001.

The generation of DAMPs during cell destruction, including the release of ATP and HMGB1, is an important indicator of immunogenic cell death. Extracellular ATP acts as a “find me” signal by activating P2RX7 receptors on DC and subsequently stimulating the priming of IFN‐γ‐producing tumor antigen‐specific CD8+ T cells.^33^ HMGB1 can induce DC maturation in a toll‐like receptor 4‐dependent manner.^34^ ATP and HMGB1 release after different focal treatments were assessed by luminescence assay and ELISA, respectively (Figure 4d,e). Cryo treatment resulted in the highest ATP (4.77 ± 0.48 μM) and HMGB1 (87.8 ± 21.1 pg/mL) release, followed closely by IRE (ATP: 3.28 ± 0.27 μM; HMGB1: 56.3 ± 11.3 pg/mL), while Heat induced the least release (ATP: 0.13 ± 0.02 μM; HMGB1: 21.8 ± 15.9 pg/mL), which aligns with the trend observed for total protein and antigen release.

### 3.3 Differential cytokine and chemokine release profiles among treatment modalities

Cytokine and chemokine levels in treated B16‐OVA cell lysates were evaluated using two complementary assays with partially overlapping analyte panels. A mouse cytokine array panel was first used to profile cytokines and chemokines in a semi‐quantitative manner. Because the array readout is based on chemiluminescent signal intensity, results were presented as relative pixel density normalized to the Cryo treatment group (Figure 5a), reflecting relative concentrations rather than absolute concentrations. To obtain precise quantitative measurements and to assess additional targets, cytokines and chemokines were further analyzed using an immunology multiplex assay, with some overlap between the two panels (Figures 5b,c and S7).

**FIGURE 5:**
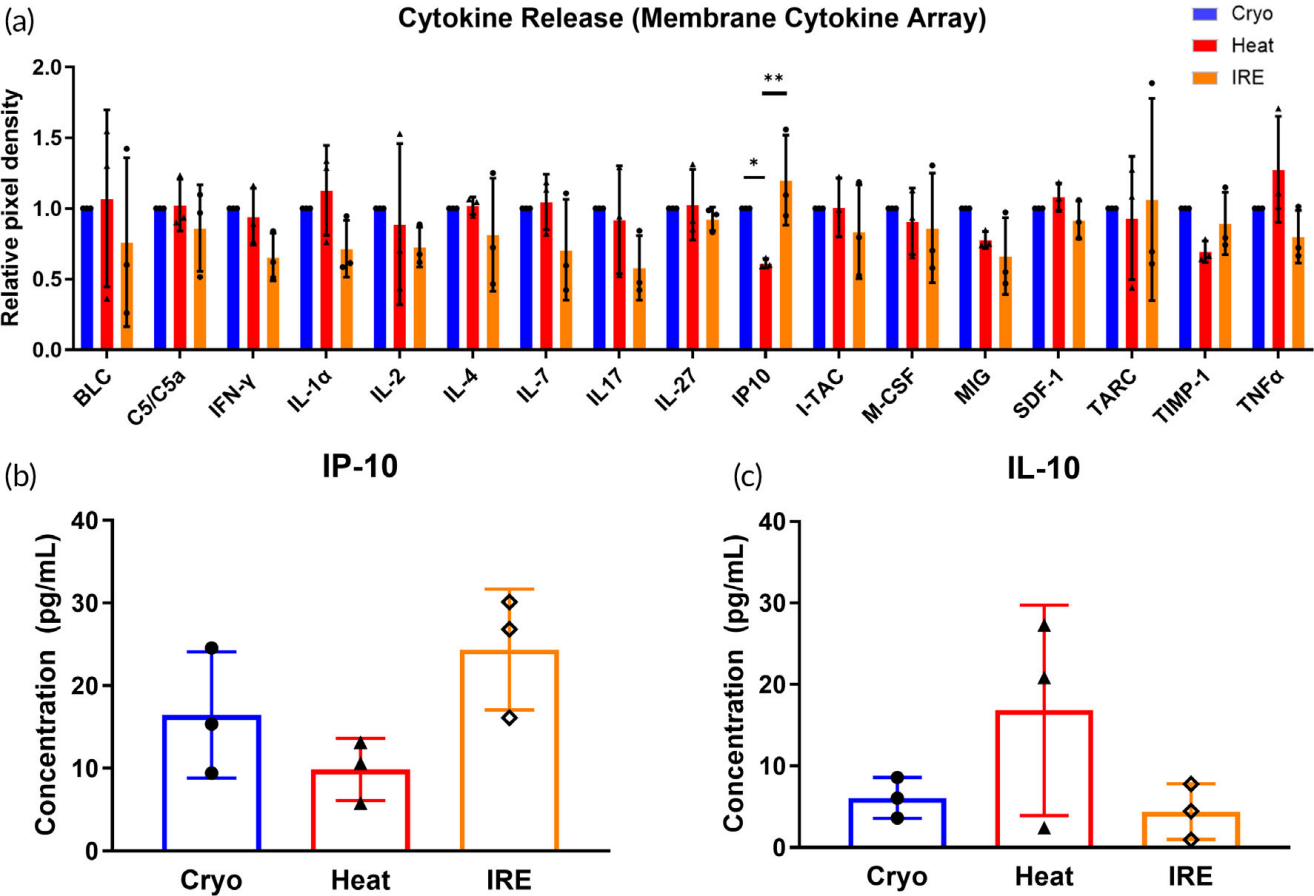
Quantification of cytokine and chemokine release from B16‐OVA cells by different focal treatments. (a) Relative concentrations of cytokine and chemokine from B16‐OVA cells by different focal treatments were measured by a cytokine array, presented as relative pixel density normalized to the Cryo treatment group. Data are presented as mean ± standard deviation (SD). Significance of differences was determined using 2‐way ANOVA with multiple‐comparison correction (Tukey's test). (b, c) Release of IP‐10 and IL‐10 from B16‐OVA cells following different focal treatments measured by an immunology multiplex assay. Significance of differences was determined using 1‐way ANOVA. *p < 0.05, **p < 0.01.

The cytokine and chemokine release profiles differed across treatments (Figures 5 and S7). IRE induced more IP‐10 release compared to Cryo (*p* = 0.4) and Heat (*p* = 0.004) (Figure 5a). The comparative trends are similar for IP‐10 (CXCL10) probed by the multiplex assay (Figure 5b). IP‐10, secreted by dying tumor cells and other innate immune cells, is a chemoattractant for activated T cells.^35^ There were noticeable differences in the release of IL‐10 among different FT conditions (Figure 5c), although the difference was not statistically significant. It was observed that B16‐OVA cells treated with IRE released the least amount of IL‐10. IL‐10 is a crucial immunosuppressive cytokine that inhibits the maturation and antigen presentation function of DCs and suppresses Th1 responses.^36^

### 3.4 Antigen presentation depends on antigen amount and exposure duration

A PE anti‐mouse H‐2K^b^ bound to SIINFEKL antibody was used to assess the antigen presentation ability of DCs after antigen feeding. The whole OVA protein undergoes processing by APCs before presentation to T cells, whereas the SIINFEKL peptide can directly be loaded onto the MHC I complex without antigen processing (Figure 6a,b). Antigen presentation, quantified by the fluorescence intensity of the bound antibody and the percentage of MHC I‐SIINFEKL complexes (defined as antigen presentation index), increased with increasing peptide and OVA concentrations, as well as with longer incubation times, indicating that antigen presentation is concentration and time‐dependent (Figures 6c,d and S8).

**FIGURE 6:**
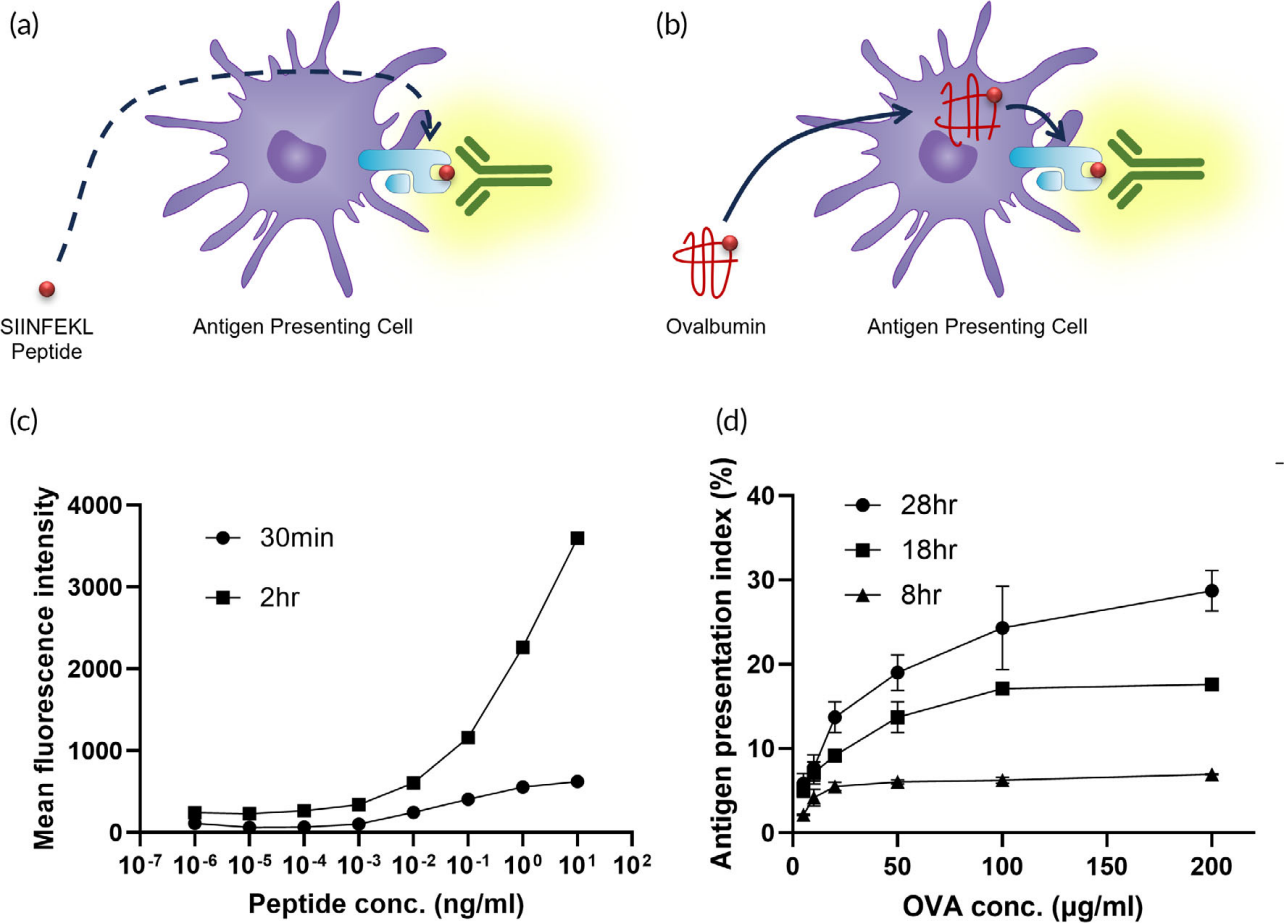
Antigen presentation of SIINFEKL peptide and OVA protein at various concentrations and incubation durations. (a) Schematic of peptide (SIINFEKL) presentation and antigen presentation tracker. (b) Schematic of antigen uptake and presentation of whole protein (Ovalbumin, OVA). (c) Antigen presentation of SIINFEKL peptide at various concentrations and different incubation durations. (d) Antigen presentation of whole OVA at various concentrations and different incubation durations.

### 3.5 Focal treatment of antigen alone did not change immune priming

To analyze the effect of focal treatments on pure antigen, antigen presentation of treated peptide and T cell activation after exposure to treated whole OVA were assessed (Figure 7a). A concentration of SIINFEKL peptide (0.01 ng/mL) was chosen for medium antigen presentation index to further compare the antigen presentation with treated peptide using different focal treatments. However, no significant differences in antigen presentation were observed between the treatment groups (Figure 7b). To quantify the extent of T cell priming, T cells were cultured with DCs fed with treated whole OVA protein, and the level of T cell activation was compared by analyzing CD44 and CD69 expression as activation markers, alongside the loss of CD62L. For mouse cells, CD69 is a typical marker for T cell activation, which peaks after only a few hours and is short‐lived.^37^ Other activation markers such as CD44 or the downregulation of CD62L are also commonly used and can last several days.^38^ The focal therapy conditions applied to pure protein (OVA) did not result in significant changes in the early activation (6 h) of antigen‐specific T cells, which is characterized by high CD44 and CD69 expression and low CD62L expression (Figure 7c‐e). In general, the presentation of antigen alone without involving tumor cells was relatively unaffected by the type of focal therapy under the conditions tested, and focal treatments on pure antigen did not cause a significant influence on immune priming, whether Heat, Cryo, or IRE.

**FIGURE 7:**
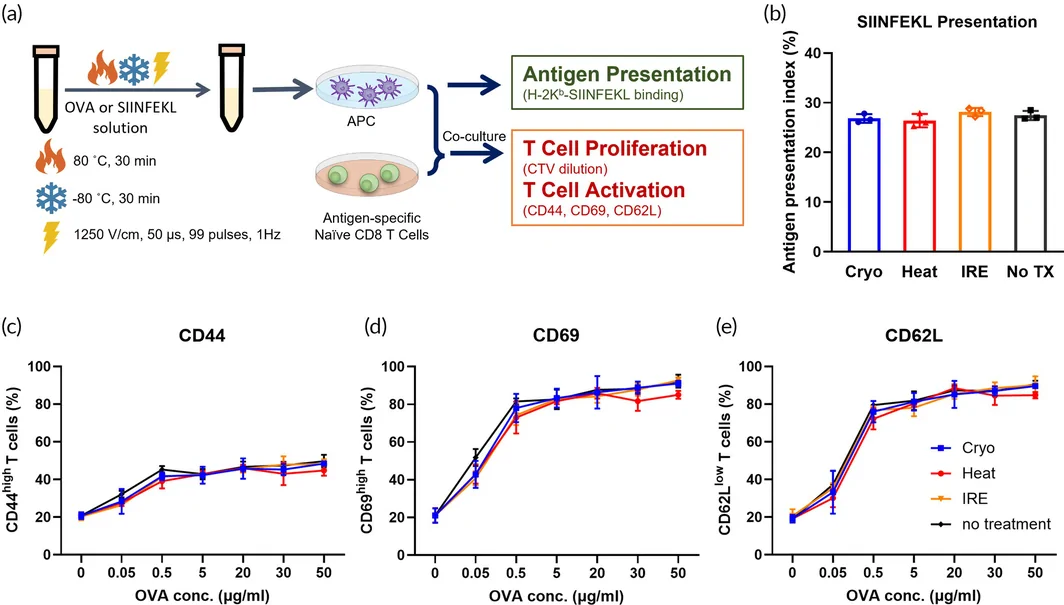
Antigen presentation of treated peptide and T cell activation after exposure to treated whole OVA. (a) Schematic of characterization assay with peptide and whole OVA. (b) Antigen presentation of SIINFEKL peptide (0.01 ng/mL) after exposure to different focal treatment conditions (Cryo, Heat, IRE or no treatment (TX)). (c) T cell early activation (6 h) following exposure to antigen (OVA)‐pulsed DCs. The DCs were exposed to OVA treated with different focal treatments for 24 h. Data are presented as mean ± standard deviation (SD).

### 3.6 Modulation of T cell and DC response by stimuli besides antigen

To investigate factors influencing immune priming beyond antigen quantity and quality, DC and T cell activation following exposure to naïve OVA and focal‐therapy‐treated B16 cell lysates were assessed (Figure 8a). B16 cells do not contain the cognate antigen specific for OT‐I T cells, however the immune priming was significantly modulated by these cell lysates. Based on the dose response across various OVA concentrations and B16 cell equivalents, a specific concentration of OVA (1 μg/mL) and a defined range of B16 cell equivalents were selected to elicit a spectrum of T cell proliferation percentage, ranging from low to high, which enables the comparison of antigen‐specific T cell response following different focal treatments. In the presence of antigen (whole OVA protein), B16 cell lysates from the various treatments significantly enhanced T cell activation and proliferation, with improvements correlating with increasing B16 cell equivalents. The IRE treatment yielded the maximum percentage (75.1 ± 15.1%) of activated and proliferated T cells (CD44^high^CTV^low^) at 1 × 10^5^ cell equivalents with 1 μg/mL OVA, followed by Cryo (52.6 ± 8.1%) and Heat (50.7 ± 8.9%) treatment (Figure 8b). Varying the concentrations of OVA, while keeping the amount of B16 cell lysates constant, does not affect DC activation (Figure S9). Therefore, a specific concentration of OVA and B16 cell equivalent was employed to induce moderate DC activation, in order to investigate whether the type of focal treatment could modulate the activation of APCs. DC activation, characterized by the elevated expression of CD86 and MHCII, was augmented following exposure to treated B16 cell lysates, with the most pronounced enhancement observed after IRE treatment of B16 cells compared to Heat (CD86: *p* = 0.02; MHCII: *p* = 0.03) and Cryo (CD86: *p* = 0.11; MHCII: *p* = 0.13) treatments (Figure 8c,d). This observation suggests a crucial role for DAMPs, cytokines and chemokines released by tumor cells and present in the cell lysates in modulating immune priming, which could contribute to the superior effect of IRE to induce highest antigen‐specific T cell proliferation when treating B16‐OVA cells.

**FIGURE 8:**
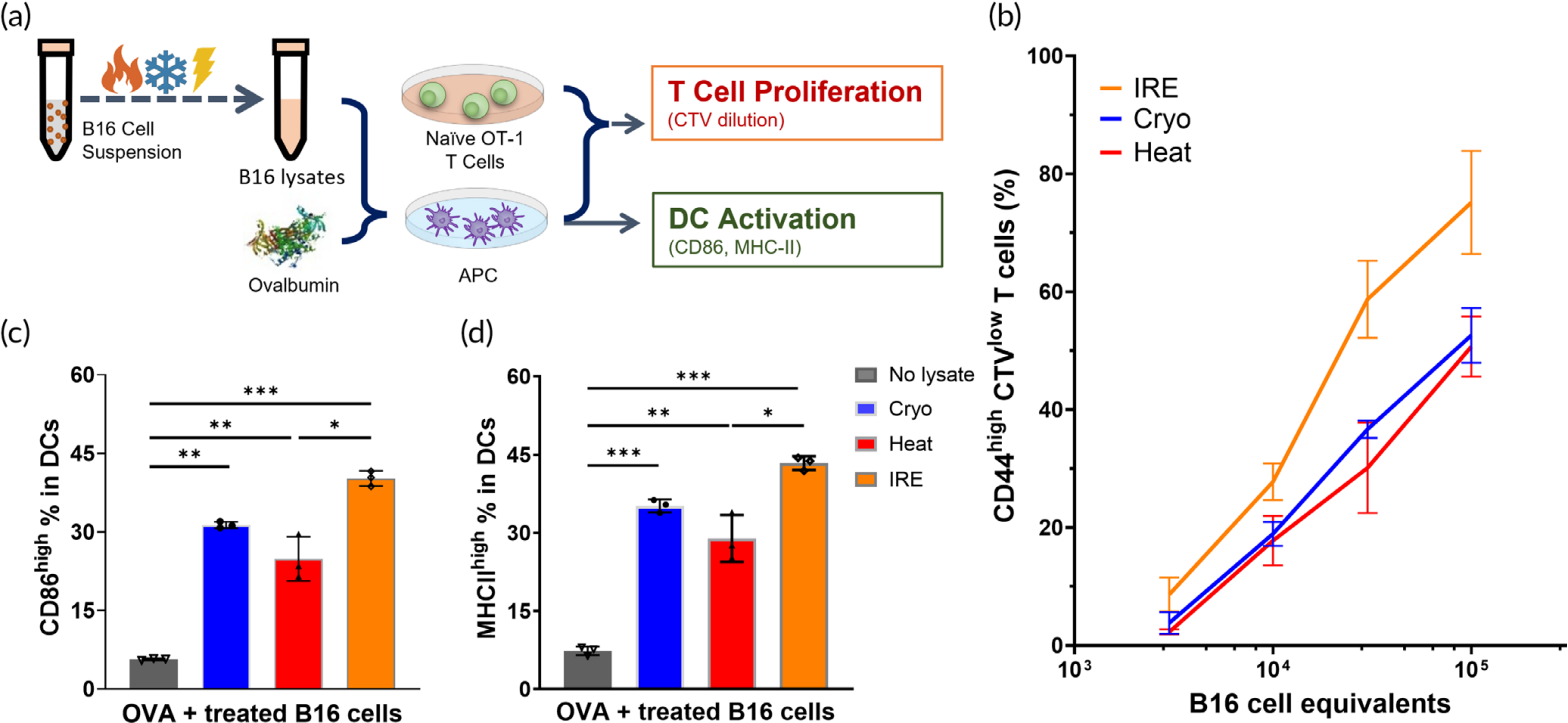
T cell and DC activation after exposure to naïve OVA in the presence of focal‐therapy‐treated B16 cell lysates. (a) Schematic of characterization assay with OVA and focal‐therapy‐treated B16 cell lysates. (b) The percentage of activated and proliferated OT‐I T cells (CD44^high^CTV^low^) in total OT‐I T cells quantified at various B16 cell equivalents. DCs were fed with naïve OVA (untreated, 1 μg/mL) and focal‐therapy‐treated B16 cell lysates for 24 h, then cocultured with OT‐I T cells for 3 days. Data are presented as mean ± standard error of mean (SEM). (c, d) Expression of DC activation marker (CD86 and MHCII) on bone marrow‐derived DCs after incubation with OVA (untreated, 5 μg/mL) and focal‐therapy‐treated B16 cell lysates (30,000 cells) for 24 h. The ratio of DCs to B16 cells was 5:1. Data are presented as mean ± standard deviation (SD). Significance of differences was determined using 1‐way ANOVA. *p < 0.05, **p < 0.01, ***p < 0.001, ****p < 0.0001.

## 4 CONCLUSION AND DISCUSSION

### 4.1 Immunogenicity is affected by focal therapeutic conditions

The characterization of T cell proliferation and DC activation reveals that the immune response to B16‐OVA cells following IRE treatment is significantly more robust compared to Cryo or Heat treatments (Figure 3), which is consistent with previous work using a B16 model characterizing TRP‐2 (tyrosinase‐related protein 2) specific response.^23^ Moreover, several studies have demonstrated that IRE can be used to effectively generate and expand tumor‐activated CTLs.^11^, ^39^, ^40^ In an in vivo study using a colorectal cancer model, IRE has a more synergistic effect with anti‐PD‐1 immunotherapy than thermal ablation or cryoablation.^41^ These findings suggest that IRE is a promising approach to induce immunogenic cell death, activate APCs, enhance antigen presentation and T cell activation, and stimulate an anti‐tumor immune response. Essentially, IRE treatment could enhance the immunogenicity of tumors, referring to their ability to elicit an immune response.

### 4.2 Comparison with previous studies

It has been well documented that the immunological effects vary significantly among FT modalities.^1^ For instance, an in vitro study has shown that the ‘quantity’ (i.e., amount) and ‘quality’ (i.e., molecular state) of tumor protein released, including antigens, can differ significantly, leading to varying degrees of priming of the immune system.^23^ Several preclinical and clinical studies also showed that the immune response, characterized by the percentage of antigen‐loaded dendritic cells (DCs) in the draining lymph node, immune cell infiltration and evaluation of inflammatory responses, varies after different ablation therapies.^42^, ^43^, ^44^

Our study utilizes the B16‐OVA model, which offers a significant advancement over the endogenous TRP‐2 model previously used.^23^ Compared to wild‐type B16 cells that rely on tumor‐associated antigens (TAAs) like TRP‐2 and gp100, genetically engineered B16‐OVA cells introduce a well‐defined model tumor‐specific antigen (TSA) that enables the tracking of the SIINFEKL peptide on the MHC‐I complex to quantify antigen processing and presentation. Crucially, our data expands upon the “quantity” hypothesis from earlier work. By supplementing lysates with naïve antigen, we isolated the adjuvant‐like properties of the IRE‐treated cell debris. We found that IRE creates a unique cytokine and DAMP profile that matures DCs more effectively than thermal or cryo‐ablated lysates, a factor that appears to outweigh simple antigen concentration. While a previous study reported that IRE released the highest quantity of protein, our findings with membrane‐bound OVA showed that Cryo resulted in the highest bulk release of protein, antigen, and ATP. This discrepancy highlights that the immunogenicity of a focal therapy is not merely a function of antigen dose, but rather depends on the biochemical nature of the cellular debris and the presence of a co‐stimulatory microenvironment.

The immunogenicity of a treated tumor is determined by its antigenicity and various immunomodulatory factors produced by both tumor cells and host cells within the tumor microenvironment.^6^, ^45^, ^46^ Antigenicity refers to the ability of tumor‐specific or tumor‐associated antigens to bind to MHC molecules, thereby initiating an immune response.^47^ This process is influenced by factors such as the expression levels of tumor antigens, the efficiency of antigen processing and presentation, and characteristics of the antigen itself, including size, molecular complexity, conformational structure, and peptide composition.^45^, ^47^ Another key aspect of immunogenicity is adjuvanticity, which facilitates the priming of antigen‐specific immune responses. Traditionally, “adjuvanticity” refers to the ability of an adjuvant (a substance that enhances the body's immune response to an antigen) to improve the efficacy of cancer treatments.^48^ In the context of immunogenicity in this article, we use the term “adjuvanticity” to describe the presence of DAMPs, which are released from or exposed on dying, stressed, or injured cells, as well as cytokines and chemokines released from tumor and immune cells following cancer treatments.^49^ These molecules and associated immunomodulatory mechanisms are important determinants of antigen presentation and T cell activation.^49^ Different focal therapies can modulate antigenicity (e.g., by altering the quantity and quality of released antigens) and adjuvanticity, thereby shaping the overall immunogenicity of the tumor.

### 4.3 Antigenicity

In this study, the amounts of antigen present in each lysate following focal therapy conditions were assessed. It was found that Cryo exposure led to the greatest amount of protein and antigen release, followed by IRE and more distantly by Heat (Figure 4). Given that exposure to low temperatures (−80 to 0 °C) generally does not induce protein denaturation, whereas electric field‐based and heat‐based tumor ablation methods can cause various extents of denaturation depending on the conditions,^24^ Cryo‐treated B16‐OVA cells are expected to release proteins that remain predominantly in their native, non‐denatured conformations. Protein denaturation can modulate antigen processing and presentation by affecting epitope accessibility, structural stability, and immunogenic recognition.^50^, ^51^ Traditionally, cryoablation is thought to induce higher post‐ablative immunogenicity compared to high‐temperature‐based methods, as thermal ablation tends to cause extensive protein denaturation and aggregation, therefore decreasing the amount of soluble protein accessible to the immune system, as well as reducing the antigenicity of protein.^7^, ^42^, ^52^, ^53^ Unlike the high temperature during thermal ablation, the freezing process maintains intact intracytoplasmic organelles and the cell ultrastructure, while opening up the plasma membrane to immune cell exposure.^7^

To evaluate the influence of protein denaturation on immune priming, we applied focal therapy conditions to pure antigen (peptide and whole protein) and assessed the DC and T cell response. However, no significant changes in antigen presentation and T cell activation were observed (Figure 7b,c). This lack of change in this study is likely related to the molecular structure of the OVA utilized. The OVA solution we employed was generated from lyophilized powder, which mostly consists of monomers. Consequently, the entire OVA may not undergo substantial conformational changes after focal treatments, as demonstrated by the analysis of its thermal properties using differential scanning calorimetry (DSC) (Figure S10). The absence of discernible peaks during the first heating cycle of both the OVA solution and OVA powder, along with the overlap between the first and second scans, suggests the absence of significant phase transitions within the examined temperature range. We also repeated the assay using ovalbumin from another vendor (EndoFit OVA, Catalog # vac‐pova, lyophilized, ≥98% purity by SDS‐PAGE) and again observed no significant differences between the focal treatments. For future studies aimed at dissecting subtle conformational changes and their impact on antigen processing and presentation, highly purified native OVA derived from egg white would be more appropriate. However, isolating native OVA at high purity requires multi‐step protein purification from a complex egg‐white matrix and represents a technically demanding and time‐intensive process.

The dose–response analysis with SIINFEKL peptide and OVA protein demonstrates that the quantity and accessibility of antigens are crucial factors for antigen presentation and T cell response (Figure 6). Without antigen uptake and processing, the SIINFEKL peptide is presented by the MHC‐I complex much more efficiently than whole OVA, with only a small amount of peptide needed (pg/ml) to induce the full proliferation of OT‐I T cells (Figure S4). During focal treatment, when cancer cells undergo predominantly necrosis, cellular contents containing antigens are released due to the loss of plasma membrane integrity, disruption of cell organelles, and degradation of nucleic acids and proteins.^1^ The antigenic material needs to be taken up by APCs, processed, and then presented to T cells. The uptake of antigens by APCs depends on several antigen‐associated properties, such as size, shape, surface charge, hydrophobicity and hydrophilicity, as well as receptor interactions.^54^ The method of focal treatment, which involves distinct cell destruction mechanisms,^1^ could impact the accessibility of antigens. For example, specific focal treatment modalities may release antigens that are more readily internalized, digested, and presented. As an electrical‐based ablation technique, IRE delivers high‐voltage electrical pulses to the tumor to cause membrane rupture, induces severe leakage of intracellular contents with less drastic alterations in protein conformation compared to thermal‐based methods, and presumably contributes to superior antigen uptake and presentation.^23^, ^27^, ^55^

### 4.4 Adjuvanticity

Besides antigen, the release of DAMPs, cytokines, and chemokines are widely studied in the immunological response to focal ablation. Here, we also quantified the release of ATP, HMGB1 and cytokines and chemokines that are important in adaptive immunity. The release profiles of HMGB1 and ATP exhibited similar trends, with Cryo inducing the most release, followed by IRE and then Heat (Figure 4). The cytokine and chemokine release profiles differed across the focal treatments but without dramatic differences, with IRE inducing more IP‐10 release and less IL‐10 release (Figures 5 and S7). Besides ATP and HMGB1, which are important indicators of immunogenic cell death, other DAMPs such as calreticulin, degradation products (e.g., DNA, RNA) and heat shock proteins also contribute to the immune response.^1^ The release profile of these uninvestigated DAMPs after IRE treatment might contribute to a more immune‐promoting environment.

In this study, we propose that IRE treatment could induce more release of immune‐promoting DAMPs, cytokines and chemokines compared to Cryo and Heat in our setting, resulting in a stronger immune response. This hypothesis is supported by the evaluation of antigen‐specific T cell response with untreated OVA and treated B16 cell lysates. B16 cell lysates following IRE treatment improved DC activation and antigen‐specific T cell proliferation compared to Heat and Cryo when the same antigen source was present (Figure 8), indicating that immune processing and T cell response can be modulated by other stimuli in cell lysates in addition to antigen. Similarly, Zhao et al. observed a significant increase in the release of DAMPs and the expression of DC activation/maturation markers in IRE‐treated KRAS cells compared to live KRAS cells.^27^ However, it is also important to recognize that DAMPs and cytokines could also contribute to the progression of cancer and promote resistance to anti‐cancer treatments. For example, variations in ATP levels (e.g., extracellular concentrations) and HMGB1 properties (e.g., concentration, redox state) can induce distinct biological responses.^56^

### 4.5 More antigen does not necessarily lead to stronger immune response

In this in vitro platform, we show that T cell activation reaches saturation at a defined protein concentration threshold (Figure 7c). Beyond this point, additional antigen does not further enhance activation and can even reduce cell viability, reflecting the limited processing and presentation capacity of antigen‐presenting cells. This non‐linear dose–response relationship is consistent with classical T cell signaling dynamics. Similar principles operate in vivo: antigen presentation saturates at physiological limits, and excessively high antigen loads can trigger regulatory pathways such as exhaustion, anergy, or tolerance.^57^, ^58^ Conversely, only small amounts of antigen are required to elicit protective immune responses, as exemplified by inactivated vaccines, where microgram‐level antigen doses are sufficient to induce robust and durable immunity.^59^

The optimal strategy for eliciting tumor‐specific immune responses is not solely dependent on antigen release: therefore, higher antigen quantity does not necessarily correlate with a stronger immunogenicity. It is crucial to avoid oversimplifying the aspects that can modulate the immune response. In this study, although IRE did not elicit the highest levels of antigen release, it emerged as the most immunogenic treatment. This finding underscores the critical role of factors beyond antigen quantity, such as antigen accessibility, the efficiency of antigen processing and presentation, and adjuvanticity, in shaping immune priming.

In clinical practice, overtreatment with ablation is common,^60^ as clinicians often target a larger tumor volume to ensure complete tumor destruction. This practice may be counter‐productive from an immunogenic perspective. While complete ablation may lead to a greater release of tumor antigens at once, it may not necessarily elicit a more effective immunogenic response. Nevertheless, if the ablation volume is excessively large and destroys the local lymphatic architecture, the ability of DCs to transport the “high‐quality” antigens we identified to the draining lymph nodes may be compromised.^61^, ^62^ This highlights the need for “precision ablation” that balances tumor destruction with the preservation of the peritumoral immune niche.

### 4.6 Limitations and future directions

While our in vitro platform successfully isolated the direct impact of focal energy fields on antigen presentation and T cell priming, we acknowledge the inherent limitations of the platform. In addition to the aspects investigated in this study, there are other biophysical factors that may influence the immune processing and immune priming, such as size distribution of lysates. It has been reported that particles in the submicron size range or less are optimal for uptake by DCs or macrophages.^22^, ^63^, ^64^ Particles ranging from 20 to 200 nm are generally taken up via endocytosis with subsequent induction of CD4 and CD8, and Th1‐type immune responses.^65^ There is also evidence that tumor‐cell‐derived exosomes contain an enriched amount of tumor antigens.^66^ The size distribution of cell lysates following different focal treatment conditions may vary significantly, and specific treatments may release more exosomes, potentially contributing to differential antigen‐specific T cell activation. Additionally, we collected supernatant as cell lysates and removed dense debris for immune analysis, which did not involve analyzing the influence of solubility and/or aggregation of cellular contents on their transportation to APCs and later processing.

Beyond the choice of focal therapeutic conditions, the physical delivery of the energy field and its parameters play a direct role in shaping the biochemical quality of the tumor lysate and therefore the immune response. For example, a recent study by Esparza et al. has demonstrated that high‐frequency irreversible electroporation (H‐FIRE) induces significant remodeling of the tumor‐draining lymphatics and microvasculature.^67^ This suggests that IRE provides a unique dual advantage: it preserves the molecular integrity of the antigens and DAMPs (as shown in our results) while simultaneously “opening” the transport pathways needed for these signals to reach the adaptive immune system. This direct effect on the stromal and immune infrastructure is a distinct advantage of pulse‐power therapies over thermal ablation, which often results in vascular and lymphatic collapse that may trap antigens within the necrotic core. It is important to note that IRE‐induced immunogenicity is highly dependent on pulse parameters. While our study utilized 50‐μs pulses to ensure non‐thermal lysis, recent studies have shown that longer pulse widths or higher frequencies can modulate the balance between apoptotic and pyroptotic death.^68^ Such variations could further refine the “quality” of the lysate and its ability to prime DCs, representing a promising area for future optimization. While our thermal ablation protocol (50°C, 30 min) was optimized for uniform in vitro cell death, clinical Radiofrequency ablation (RFA) or microwave ablation (MWA) parameters involve higher temperatures and shorter durations. Because the thermal dose (time vs. temperature) influences the degree of protein coagulation and DAMP preservation, future studies should investigate how varying the “speed” of thermal ablation impacts the subsequent immune cell response compared to the non‐thermal mechanism of IRE.

While the OVA model is powerful, it does not fully recapitulate the nature of endogenous tumor antigens in terms of expression level, immunogenicity, and immune pathway. Moreover, the in vitro analysis platform cannot replicate the tumor environment, which includes many types of immune cells and complex cell–cell interactions, as well as the recruitment or migration patterns of these cells. Additionally, focal ablation of tumors induces a tissue injury response and initiates the wound healing process, which engages both the adaptive and innate immune systems.^6^, ^69^, ^70^ Further investigation using in vivo models and detailed profiling of antigen processing, antigen presentation, and antigen‐specific T cell response is necessary to deepen our understanding of the immunogenic aspects of focal therapy. Furthermore, robust clinical evidence demonstrating the antitumor immune response induced by focal therapy is warranted.

We acknowledge the inherent limitations of translating these findings directly to the clinic. The in vitro setting lacks the complex immunosuppressive landscape of the tumor microenvironment, which includes regulatory T cells and myeloid‐derived suppressor cells that can attenuate the robust T cell proliferation observed in our assays. Furthermore, clinical focal ablation induces secondary effects such as vascular disruption and systemic inflammatory cascades that are not captured in a cell culture model. However, by simplifying the system, we have demonstrated that the fundamental “quality” of the immunogenic signal generated by IRE is more immunogenic under the conditions tested to that of thermal modalities. Future in vivo studies will be essential to determine if this mechanistic advantage is sufficient to overcome the physical and biological barriers present in a syngeneic tumor model.

## AUTHOR CONTRIBUTIONS

**Minhan Jiang:** Writing – original draft; conceptualization; methodology; investigation; validation; formal analysis; data curation; visualization. **Qi Shao:** Writing – original draft; conceptualization; data curation; investigation; validation; resources; project administration; writing – review and editing; funding acquisition; methodology. **John Bischof:** Funding acquisition; conceptualization; validation; supervision; project administration; resources; writing – review and editing. **Brandon Burbach:** Conceptualization; methodology; data curation; investigation; validation; writing – review and editing; formal analysis. **Yoji Shimizu:** Funding acquisition; resources; supervision. **Samira Azarin:** Funding acquisition; conceptualization; supervision; project administration. **Joseph Vallin:** Methodology; data curation; validation; visualization; resources.

## FUNDING INFORMATION

This study was funded by Boston Scientific Corporation (Maple Grove, MN), Dr. Ralph and Marian Falk Medical Research Trust Bank of America, and the Medtronic‐Bakken Endowed Chair for Engineering in Medicine (JB) at University of Minnesota.

## CONFLICT OF INTEREST STATEMENT

The authors declare no conflicts of interest.

## ETHICS STATEMENT

This study utilized commercially available murine cell lines and primary cells from live mice. All animal usage and experimental procedures were reviewed and approved by the Institutional Animal Care and Use Committee (IACUC) of the University of Minnesota. No other live animal or human subjects were used in this specific in vitro study.

## Supporting information


**FIGURE S1.** Dendritic cell (DC) phenotyping after 9 days differentiation with Flt3 ligand.
**FIGURE S2.** Flow cytometry gating strategy for DC activation.
**FIGURE S3.** Flow cytometry gating strategy for T cell activation.
**FIGURE S4.** T cell proliferation in the absence of antigen, with SIINFEKL peptide, or with whole OVA.
**TABLE S1.** Comparison of T cell proliferation following exposure to focal therapy treated B16‐OVA cell lysates using the 2way ANOVA with Turkey's multiple comparisons.
**FIGURE S5.** Expression of DC activation markers (CD80 and MHCII) after exposure to treated B16‐OVA cell lysates.
**FIGURE S6.** Representative western blot from independent B16‐OVA lysate preparations.
**FIGURE S7.** Quantification of cytokine release in B16‐OVA lysates by different focal therapies using an Immunology Multiplex Assay.
**FIGURE S8.** Flow cytometry analysis of H‐2K^b^‐SIINFEKL antibody binding at varying peptide concentrations.
**FIGURE S9.** DC activation after exposure to focal therapy treated B16 cell lysates with varying concentration of OVA.
**FIGURE S10.** Differential scanning calorimetry (DSC) thermal analysis of OVA solution (1 mg/mL) and OVA powder (5 mg).

## Data Availability

The data that support the findings of this study are available from the corresponding author upon reasonable request.
