# Supplementary material for: Focal therapeutic conditions matter: How energy affects antigen presentation and T cell activation
Source: Bioeng Transl Med. 2026 May 27;11(5):e70152. doi: 10.1002/btm2.70152 (PMC13636184; doi:10.1002/btm2.70152)
Supplement: Supplementary file 1 [file BTM2-11-e70152-s001.docx]

Focal Therapeutic Conditions Matter: How Energy Field on Cancer Cells Affects Antigen Presentation and T Cell Activation

**Supplemental Information**

Figure S1. Dendritic cell (DC) phenotyping after 9 days differentiation with Flt3 ligand.

Figure S2. Flow cytometry gating strategy for DC activation.

Figure S3. Flow cytometry gating strategy for T cell activation.

Figure S4. T cell proliferation in the absence of antigen (first row), with SIINFEKL peptide (second row), or with whole OVA (third row).

| Cell equivalents |  | P value |  | P value |
| --- | --- | --- | --- | --- |
| 100000 | Cryo vs. IRE | 0.0076 | Heat vs. IRE | 0.0059 |
| 200000 | Cryo vs. IRE | <0.0001 | Heat vs. IRE | <0.0001 |
| 500000 | Cryo vs. IRE | 0.0002 | Heat vs. IRE | <0.0001 |

Table S1. Comparison of T cell proliferation following exposure to focal therapy treated B16-OVA cell lysates using the 2way ANOVA with Turkey’s multiple comparisons. The comparisons between Cryo, Heat and IRE treatments with p < 0.05 are shown here.

Figure S5. Expression of DC activation markers (CD80 and MHCII) after exposure to treated B16-OVA cell lysates. Data are presented as mean ± standard deviation (SD). Significance of differences was determined using 2-way ANOVA. *p < 0.05, **p < 0.01, ***p < 0.001.

Figure S6. Representative western blot from independent B16-OVA lysate preparations (one of three biological replicates). Molecular weight markers (kDa) are shown alongside the bands.

Figure S7. Quantification of cytokine release in B16-OVA lysates by different focal therapies using an Immunology Multiplex Assay. MILLIPLEX MAP Mouse Cytokine/Chemokine Magnetic Bead Panel - Premixed 25 Plex was used. Data are presented as mean ± standard deviation (SD).

Figure S8. Flow cytometry analysis of H-2K^b^-SIINFEKL antibody binding at varying peptide concentrations. Antigen-presenting cells (APCs) were pulsed with SIINFEKL peptide at a series of concentrations for 2 hours. Antigen presentation by dendritic cells was assessed using PE-conjugated anti–mouse H-2Kᵇ bound to SIINFEKL. Presentation levels were quantified by the fluorescence intensity of the bound antibody.

Figure S9. DC activation after exposure to focal therapy treated B16 cell lysates with varying concentration of OVA. Bone marrow-derived DCs were incubated with various concentrations of OVA and focal therapy treated B16 cell lysates (30000 cells) for 24 hr. The ratio of DCs to B16 cells was 5:1. Data are presented as mean ± standard deviation (SD). Significance of differences was determined using 2-way ANOVA. *p < 0.05, **p < 0.01, ***p < 0.001, ****p < 0.0001.

Figure S10. Differential scanning calorimetry (DSC) thermal analysis of OVA solution (1 mg/mL) and OVA powder (5 mg). Samples were heated from 20°C to 90°C, cooled to 20°C, reheated to 90°C, and cooled back to 20°C at a rate of 5°C per minute.
